# Current Understanding of Food Safety Assessment Procedure for Stacked Trait Products Derived from Conventional Breeding among Approved GM Plants

**DOI:** 10.14252/foodsafetyfscj.D-20-00003

**Published:** 2020-03-27

**Authors:** Wataru Iizuka

**Affiliations:** Food Safety Commission of Japan Secretariat, Akasaka Park Bldg., 22F, Akasaka 5-2-20, Minato-ku, Tokyo 107-6122, Japan

**Keywords:** GM plants, food safety assessment, stacked trait products

## Abstract

Ministry of Health, Labour and Welfare (MHLW) has conducted food safety assessment of the genetically modified (GM) plants generated by conventional breeding among approved GM plants (hereinafter called “stacked trait products”). Food safety assessment procedure of stacked trait products was revised in 2014. The safety assessment policies will continue to be updated, based on the collected knowledge and experience.

## 1. Introduction

After the introduction of genetically modified (GM) plants, the Ministry of Health, Labour and Welfare (MHLW) started to conduct the food safety assessment in Japan. After the establishment of Food Safety Commission of Japan (FSCJ) as a risk assessment body in accordance with the Food Safety Basic Law established in 2003, the MHLW continues to evaluate the safety of GM plants as a risk management body by way of requesting a risk assessment to the FSCJ. Based on accumulated experiences of 10 years under the current administration system in food safety assessment, the MHLW revised the procedure for food safety assessment of the GM plants generated through conventional breeding of approved GM plants in which no influences are observed on the metabolisms of the host plants by the transgenes (hereinafter called “Type 1 × Type 1 stacked trait products”) in 2014.

In this context, development of food safety assessment system of GM plants and the recent revision in Japan are briefly summarized focusing on the stacked trait products.

## 2. Overview of Safety Assessment of Stack Varieties

The MHLW assesses the safety of stacked trait products through consultations with the FSCJ for each application, according to the Ministerial Notification No. 233 in 2000 entitled “Procedures of Application for Safety Assessment of Foods and Food Additives Produced by Recombinant DNA Techniques”. Parental single GM plants that are crossed to produce stacked trait products are classified into the following three categories, Type 1, Type 2 and Type 3 as follows, according to the introduced traits in FSCJ’s food safety assessment.

Categories of GM plants as;

1) Type 1: GM plants with traits, such as herbicide tolerance, insect resistance or virus resistance, in which no influences are observed on the metabolisms of the host plants by the transgenes.

2) Type 2: GM plants with traits, such as increased amounts of nutritional components, or altered content of cell wall components, in which metabolic pathways of the host plants are altered by the inserted genes.

3) Type 3: GM plants with “de novo generated substances”, in which new substances are produced by the inserted genes, using metabolites of the host plants.

The FSCJ published “Stance on the Safety Assessment of GM Plants Generated through Cross-Breeding^1^^)^” in 2004. In the stance, all the stacked trait products are noted to be assessed in considering the combination of types (e.g. Type 1 × Type 1, Type 1 × Type 2, Type 2 × Type3), irrespective of the number of conventional breeding events. It was also noted that the safety assessment of GM plants generated through the crossings of Type 1 x Type 1 stacked trait products is necessary for the time being in the following cases. These contain (1) the GM plants generated through crossing among different subspecies or the higher taxonomic levels and (2) the changes in the amounts of dietary intakes, edible parts, and/or processing methods, etc., are intended.

## 3. Revised Concept of Safety Evaluation of Type 1 × Type 1 Stacked Trait Products

Safety assessment of GM plants generated through cross-breeding of GM plants had been conducted for 10 years until the revision in 2014, and no influences are observed on the metabolisms of the host plants by the transgenes (i.e. Type 1 × Type 1 stacked trait products). With the accumulations of the data and experiences, assessment procedure for stacked trait products was reviewed by the MHLW in 2014. The MHLW subcommittee concluded that the safety assessment is no longer required for each of Type 1 × Type 1 stacked trait products. These are recognized to be fallen into the category, just like the progeny cultivars^1^ which are generated through conventional cross-breeding between the approved GM and non-GM plants.

Then the MHLW’s Ministerial Notification No. 233 was revised to reflect the conclusion of the subcommittee. This conclusion was based on the following rationales^2^^)^:

1) Safety of parental GM plants is confirmed by FSCJ’s safety assessment, and presence/absence of influences on the metabolisms of the host plants by the transgenes are predictable from the safety assessment of each parental GM plant.

2) Total 191 cases of assessments were conducted on Type 1 × Type 1 stacked trait products (as of October 17, 2013). These suggest no apparent alterations on bio-efficacies among products of the traits of parents GM plants and the stacked trait of this category during the cross-breeding.

**Fig. 1.**
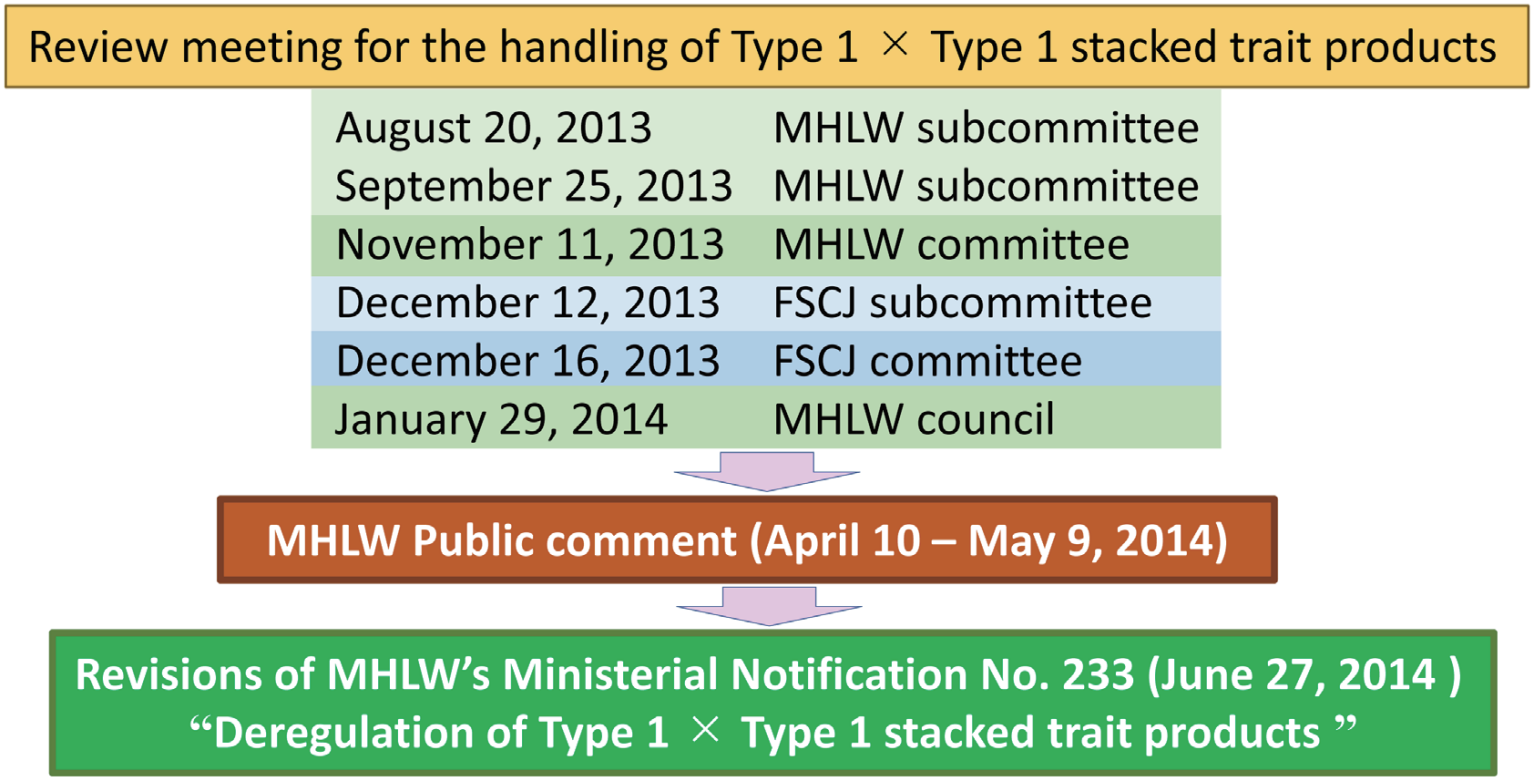
Administrative process for deregulation of Type 1 × Type 1 stacked trait products

## 4. Changes after the Revised MHLW’s Ministerial Notification No. 233 (2014)

Total 106 events of GM plants have been approved until September 2019, and 98 events among them are categorized into GM plants that do not alter metabolic pathways of the host plant (i.e. Type 1 event). Although Type 1 × Type 1 stacked trait products are no longer required to have the food safety assessment, the developers of the Type 1 × Type 1 stacked trait products have to report the commercial plans on each of the products individually to MHLW from the standpoint of safety management.

After the revision of MHLW’s Ministerial Notification No. 233 in 2014, no assessment has been conducted for stacked trait products. These fall into Type 1 × Type 1, independent of the number of Type 1 events combined. The assessment and administrative process for Type 1 × Type 1 stacked trait products has been greatly reduced.

## 5. Recent Progress in Safety Evaluation of Type 1 x Type 1 Stacked Trait Products

At present, safety assessment is conducted only in the following case. The GM plants, classified into Type 1 x Type 1 stacked trait products, are generated through crossing among different subspecies or the higher taxonomic levels. Accordingly, GM plants obtained through cross-breeding of cotton (*Gossypium hirsutum*) and pima cotton (*Gossypium barbadense*) were assessed about the safety. Cotton and pima cotton are classified as different species of the same cotton genus, but both have double chromosome structures with high genetic similarity and can be hybridizable to each other in the field environment. In fact no differences are detected in intake amounts, processing methods, ingestion site, harmful and/or physiologically active substances, etc., and they are recognized to be the same species in the safety assessment as GM plants. Thus, an additional paper to “Stance on the Safety Assessment of GM Plants Generated through Cross-Breeding” was published by FSCJ on November 13, 2019^3^^)^. In the paper, the procedure of the safety review is noted as unnecessary in GM plants obtained through cross-breeding of cotton and pima cotton.

## 6. Conclusion

In Japan, the MHLW is a risk management body to evaluate the safety of GM plants and to request a risk assessment of those plants from the FSCJ. Under this regulatory system, the MHLW and the FSCJ revised the procedure for safety assessment of the GM plants generated through cross-breeding of approved GM plants. They decided that the safety assessment is no longer necessary for each of Type 1 × Type 1 stacked trait products which are apparent to be fallen into this category, based on the knowledge and accumulated experiences of food safety assessment, in the revision of MHLW’s Ministerial Notification No. 233 in 2014. Of course, the safety assessment policies will continue to be updated, based on the collected knowledge and experience, from now on as well.

## Disclaimer notice

The views and opinions expressed in the paper are those of the author and should not be attributed to the MHLW or the FSCJ.

## Footnote

1. The GM plants generated through conventional cross-breeding between the approved GM and non-GM plants are defined as “progeny cultivar through crossbreeding”. The progeny cultivars through crossbreeding are regarded as the safety confirmed GM plants when the following conditions are fulfilled. i) Traits newly acquired through recombinant DNA technique have not altered in progeny cultivar, ii) No cross-breeding between subspecies and iii) Neither the dietary intake, edible part nor processing methods, etc. is changed.

## References

[1] Food Safety Commission of Japan. Stance on the Safety Assessment of GM Plants Generated through Cross-Breeding. http://www.fsc.go.jp/hyouka/index.data/GM_plants_through_cross-breeding.pdf. Published January 29, 2004.

[2] Ministry of Health, Labour and Welfare. Pharmaceutical Affairs and Food Sanitation Council. https://a.msip.securewg.jp/docview/viewer/docNA18BCD693C6797a9e055df28f723e81eb047440acfe77672d5ef12469b15ea2fb5d8e43a2a67. Published November 11, 2013.

[3] Food Safety Commission of Japan. Stance on the Safety Assessment of GM Plants Generated through Cross-Breeding (Interpretation of (1), a) for the time being). http://www.fsc.go.jp/senmon/idensi/index.data/gm_kangaekata_kaishaku.pdf. Published November 13, 2019.

